# Unsupervised Deep Learning for Advanced Forming Limit Analysis in Sheet Metal: A Tensile Test-Based Approach

**DOI:** 10.3390/ma16217001

**Published:** 2023-11-01

**Authors:** Aleksandra Thamm, Florian Thamm, Annette Sawodny, Sally Zeitler, Marion Merklein, Andreas Maier

**Affiliations:** 1 Pattern Recognition Lab, Friedrich-Alexander-Universität Erlangen-Nürnberg, Martensstr. 3, 91058 Erlangen, Germany; florian.thamm@fau.de (F.T.); sally.zeitler@fau.de (S.Z.); andreas.maier@fau.de (A.M.); 2 Institute of Manufacturing Technology, Friedrich-Alexander-Universität Erlangen-Nürnberg, Egerlandstr. 13, 91058 Erlangen, Germany; annette.sawodny@fau.de (A.S.); marion.merklein@fau.de (M.M.)

**Keywords:** forming limit curve, pattern recognition, sheet metal forming, machine learning

## Abstract

An accurate description of the formability and failure behavior of sheet metal materials is essential for an optimal forming process design. In this respect, the forming limit curve (FLC) based on the Nakajima test, which is determined in accordance with DIN EN ISO 12004-2, is a wide-spread procedure for evaluating the formability of sheet metal materials. Thereby the FLC is affected by influences originating from intrinsic factors of the Nakajima test-setup, such as friction, which leads to deviations from the linear strain path, biaxial prestress and bending superposition. These disadvantages can be circumvented by an alternative test combination of uniaxial tensile test and hydraulic bulge test. In addition, the forming limit capacity of many lightweight materials is underestimated using the cross-section method according to DIN EN ISO 12004-2, due to the material-dependent occurrence of multiple strain maxima during forming or sudden cracking without prior necking. In this regard, machine learning approaches have a high potential for a more accurate determination of the forming limit curve due to the inclusion of other parameters influencing formability. This work presents a machine learning approach focused on uniaxial tensile tests to define the forming limit of lightweight materials and high-strength steels. The transferability of an existing weakly supervised convolutional neural network (CNN) approach was examined, originally designed for Nakajima tests, to uniaxial tensile tests. Additionally, a stereo camera-based method for this purpose was developed. In our evaluation, we train and test materials, including AA6016, DX54D, and DP800, through iterative data composition, using cross-validation. In the context of our stereo camera-based approach, strains for different materials and thicknesses were predicted. In this cases, our method successfully predicted the major strains with close agreement to ISO standards. For DX54D, with a thickness of 0.8 mm, the prediction was 0.659 (compared to ISO’s 0.664). Similarly, for DX54D, 2.0 mm thickness, the predicted major strain was 0.780 (compared to ISO 0.705), and for AA6016, at 1.0 mm thickness, a major strain of 0.314 (in line with ISO 0.309) was estimated. However, for DP800 with a thickness of 1.0 mm, the prediction yielded a major strain of 0.478 (as compared to ISO 0.289), indicating a divergence from the ISO standard in this particular case. These results in general, generated with the CNN stereo camera-based approach, underline the quantitative alignment of the approach with the cross-section method.

## 1. Introduction

The automotive industry engages lightweight materials, such as aluminum alloys and high-strength steels for body parts, to minimize vehicle weight and subsequently mitigate CO${}_{2}$ emissions during vehicle operation. For a resource-efficient production of such components, a simulative component design with high prediction quality based on precisely defined mechanical parameters and the exact description of the formability as input parameters is decisive. The material behavior of lightweight materials is distinguished by their mechanical values, diminished ductility and higher strength, which results in this respect in the challenge of an exact description of the formability and failure behavior of these materials [1].

The forming limit curve (FLC), which is a graphical representation of the forming limit under different strain conditions, developed by Keeler [2] and Goodwin [3], has gained significant recognition and widespread adoption in the industry for formability evaluation. The process of determining the forming limit curve is inter alia currently standardized in DIN EN ISO 12004-2 [4]. Accordingly, the Nakajima test [5] or Marciniak test [6] have to be conducted to establish the experimental data basis. For companies, this incurs additional costs as it necessitates an additional testing facility and significant material expenses due to the requirement of testing various specimen geometries to cover the complete forming limit curve. Furthermore, contrary to the linear strain paths assumption specified in the standard, the Nakajima test results in corresponding deviations caused by the mutually influence of tribological properties between the punch and the sheet, biaxial prestress, and the observed bending effect when the sheet is arched upwards. To counteract these disadvantages and offer a cost-effective approach for determining the forming limit curve, Kohl et al. [7] proposed an alternative test combination consisting of the uniaxial tensile test, plane-strain test and hydraulic bulge test (HBT). The calculation of the support points of the FLC based on these tests is carried out according to the evaluation method specified in the DIN EN ISO 12004-2 standard, the cross-section method. This procedure encompasses the fitting and evaluation of an inverse parabolic curve to the experimentally in characterization tests defined strain distribution prior to material fracture. The strains over the specimen surface are calculated using Digital Image Correlation (DIC). In relation to the strain distribution over the specimen, the application of the cross-section method leads to a material-dependent evaluation of the forming limit. Thus it is well suitable for materials exhibiting significant necking behavior, such as deep-drawing steels, but it tends to yield more understated estimates of forming limits for lightweight materials that exhibit abrupt cracking or the presence of multiple strain maxima. Additionally, this method approximates the forming limit using a parabolic fit at a defined area, which limits the inclusion of all the provided information in the evaluation process. Machine learning algorithms provide the potential to enhance the prediction accuracy of forming limits by incorporating additional factors that influence failure behavior and identify non-obvious relationships among them. Existing approaches have already demonstrated a good prediction of the forming limit curve by using feedforward backpropagation artificial neural networks (NN). These networks utilize input data obtained from various sources, including process-related data from test trials [8,9], geometric data of the test specimen [10], and calculated stress-strain values derived from material testing [11,12,13]. Other authors obtained results in good agreement with the respective reference points using adaptive network fuzzy inference systems (ANFIS). The input is given by strain values [14] and additional microstructure describing features and process parameters [15]. The input data of the previously mentioned approaches are based on location-independent input streams. Since spatial relationships between different features over the specimen surface arise during the forming process, Jaremenko et al. [16] developed an approach that incorporates these dependencies. Three-channel gray scale images are generated from the numerical description of the strain distribution over the specimen surface in the Nakajima test using x- and y-coordinates, as well as the three channels major strain, minor strain and thickness reduction. The resulting video sequence is used as input for various machine learning algorithms, support vector machines [16] and Deep Learning (DL) NN [17]. The classification result categorizes the forming process into distinct phases: homogeneous forming, diffuse necking, local necking and crack. Subsequently, a probabilistic-based evaluation method is employed to evaluate this result in terms of determining the forming limit. For all approaches a good prediction accuracy is obtained. Especially the weakly supervised DL approach with Siamese architecture differs from the other approaches due to its independence with respect to the cracking location and the cracking time and the advantage of not having to rely on an expert annotation, which keeps the effort in the training phase low [16].

In pursuit for an innovative approach to efficiently determine the forming limit curve, one that can effectively address intricate strain distributions with multiple strain maxima and sudden cracking, this research significantly improves upon prior methodologies. The objective is to assess the transferability of their approach to alternative forming limit curve (FLC) testing, with a primary focus on tensile tests, applied to deep-drawing steel (DX54D), dual-phase steel (DP800), and an aluminum alloy (AA6016). In contrast to existing methodologies, this work introduces a novel approach that eliminates the need for labor-intensive strain value calculations using Digital Image Correlation (DIC). The distinctive innovation of this study lies in its departure from conventional techniques and the integration of advanced image-based technology to predict FLC points. Instead of DIC, it leverages only images captured by a stereo camera system as an input, significantly reducing the complexity involved in constructing the training set. By analyzing the network’s performance across diverse materials, this research seeks to demonstrate its ability to generalize and effectively handle unforeseen input scenarios. A crucial benchmark for assessing the accuracy and reliability of the results generated by the network is provided by a reference point established through the cross-section method.

## 2. Materials and Experimental Setup

### 2.1. Materials

**Figure 1:**
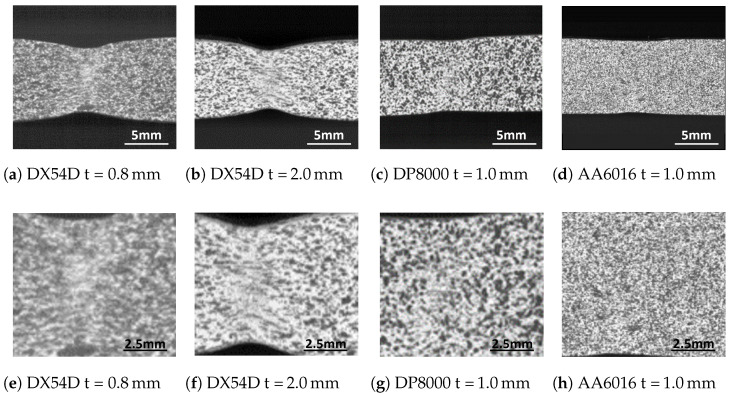
Local necking in the frame before fracture of DX54D (UE = 22%), DP800 (UE = 11%) and AA6016 (UE = 17%).

Three different materials, a deep-drawing steel DX54D, a dual-phase steel DP800 and an aluminum alloy AA6016 are investigated in this study. A summary of the mechanical properties and chemical composition of the materials can be found in Table 1 and Table 2. The deep-drawing steel is a low-alloy steel material with a thicknesses of 0.8 mm and 2.0 mm. It is characterized by a high forming ability up to the beginning of local necking, as well as a significant necking phase (Figure 1e,f) and thus a high elongation at fracture. In addition to its good formability, the material is resistant to aging and is therefore used for the production of sophisticated outer and inner body parts.

The dual-phase steel DP800 with a thickness of 1.0 mm belongs to the group of high-strength steels. The material has a high yield strength. Due to the inhomogeneous structure of ferrite and martensite, it exhibits multiple strain maxima, sudden fracture initiation and no significant necking phase (Figure 1) during forming [18]. Due to the high strength of the material, it is used for safety-relevant structural components. The aluminum alloy AA6016 with a thickness of 1.0 mm, like all aluminum alloys of the 6000 class, is characterized by corrosion resistance and freedom from strecher strain marks. Therefore, it is used in the automotive industry especially for structural and outer body components.

### 2.2. Experimental Setup

**Figure 2:**
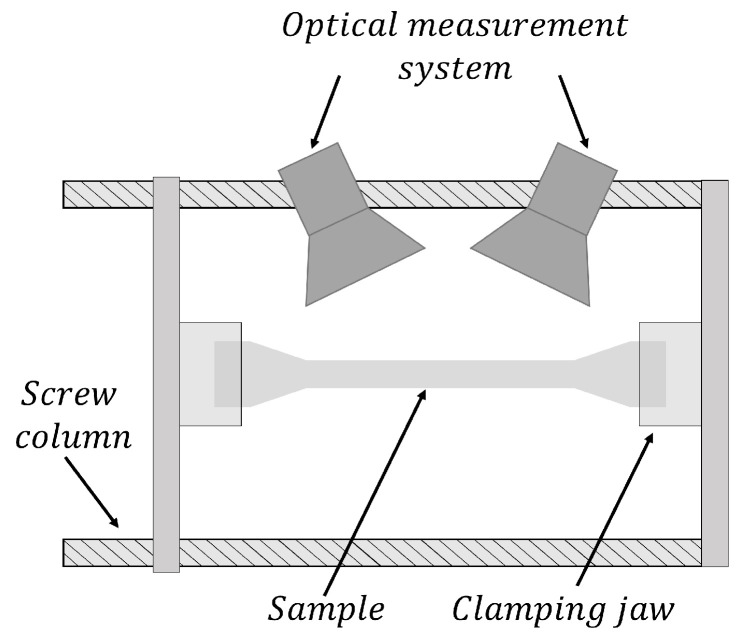
Schematic setup of the tensile tests, including the stereo camera system.

For the calculation of the support point of the forming limit curve under uniaxial loading condition, tensile tests according to DIN EN ISO 6892-1 [19] were performed on a Z100 universal testing machine from Zwick AG (Ulm, Germany). A specimen geometry according to the DIN EN ISO 6892-1 standard with an initial length of 50 mm was selected and manufactured by laser cutting with subsequent edge processing carried out by milling. Prior to the tensile test, the evaluation area was coated with a stochastic black graphite pattern on a white background. The tensile specimens were drawn until different timepoints of the forming process (Table 3), applying a constant strain rate of 0.4%/s for steel and 0.667%/s for aluminum alloys. Loading was performed along the preferred failure direction, with 90° to the rolling direction for steel and 0° for aluminum alloys. The forming process was recorded with the 3D optical measuring system Aramis from GOM GmbH (Braunschweig, Germany) at a recording frequency of 30 Hz and evaluated using the corresponding GOM software Aramis Professional 2020 by DIC. The test setup and geometry are shown in Figure 2. In addition to the optical measuring system, a load cell records the force curve over time. Together with the traverse path, this was transmitted to the Aramis software via an analog input and is used as the basic data for determining the stress-strain diagram.

## 3. Method

In this research, we chose to use a Convolutional Neural Network (CNN) approach for several reasons. CNNs have shown great proficiency in tasks related to image and pattern recognition. One key advantage is that a convolutional layer, by design, processes the data in a way that aligns with our understanding of the problem. It uses kernels to compare local measurements related to fracture and contrasts them with the surrounding context. Additionally, the convolutional layer’s increasing receptive field enables it to perform increasingly complex comparisons, ultimately providing the feature space necessary for the final prediction. Forming limit analysis involves identifying complex patterns and transitions in the forming process, particularly the emergence of localized necking. CNNs are well-suited to learn and discern such intricate patterns, making them a strong candidate for this application.

**Figure 3:**
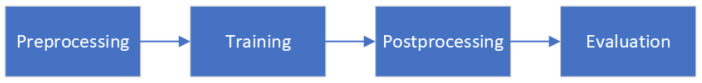
Overview of the four fundamental steps in both numerical and image-based approaches.

Both developed approaches, the numerical and image-based essentially comprise four key steps: preprocessing, training, postprocessing, and evaluation (see Figure 3). The first step, preprocessing, is primarily concerned with preparing raw data for analysis. This involves tasks such as data cleaning, structuring, and ensuring data consistency to establish a suitable foundation for subsequent analysis. During the training phase, the system acquires an understanding of patterns and relationships present within the preprocessed data. The postprocessing step focuses on extracting relevant features or insights from the model’s output. The last part, the evaluation is about how well the system or model is performing. For a comprehensive description of all four phases for both approaches, please refer to the details provided below.

### 3.1. Numerical Approach

For the numerical approach, grey scale images are generated based on the three channels: major strain, minor strain and material thickness. The classification output refers to the time point in the video sequence at which a forming phase begins or ends. Data processing is based on the processing pipeline developed by Jaremenko et al. [17]. The preprocessing of the data is based on the Aramis software version v6.3.0-7. By using the version of Aramis Professional 2020 the structure of the output data changes, which makes an adaptation of the step necessary. The other steps can be transferred to the present use case.

#### 3.1.1. Preprocessing

As mentioned Jaremenko et al. [17], who developed on the software Aramis (v6.3.0-7), where the strains were computed via rectangle facets. The data was represented as a matrix where each matrix element corresponds to a data point, and each data point $a_{i,j}$ can be uniquely identified by its index pair i,j. In the new version of the Aramis software, the strains are measured in hexagonal deformation with no matrix/grid-like structure is present, which meaning no indices exist. To use DL methods on non-indexed grids, the data has been interpolated into an indexed grid. In this work, the data has been linearly interpolated (with a grid size of 200×200). The fineness of the interpolated grid can also vary arbitrarily. To reduce the data to the relevant content a window with height and length of 40×40 is cropped from the center.

#### 3.1.2. Training

The Siamese CNN includes two identical sub-networks. Each consists of the first three convolutional blocks of a VGG16 network provided by Simonyan et al. [20], pre-trained on a large-scale image database according to Deng et al. [21]. To counteract overfitting the Siamese network includes one dropout layer (0.5 dropout rate) between the two fully connected layers (512, 256 neurons) followed by one L2 normalization layer, in line with Jaremenko et al. [17].

**Figure 4:**
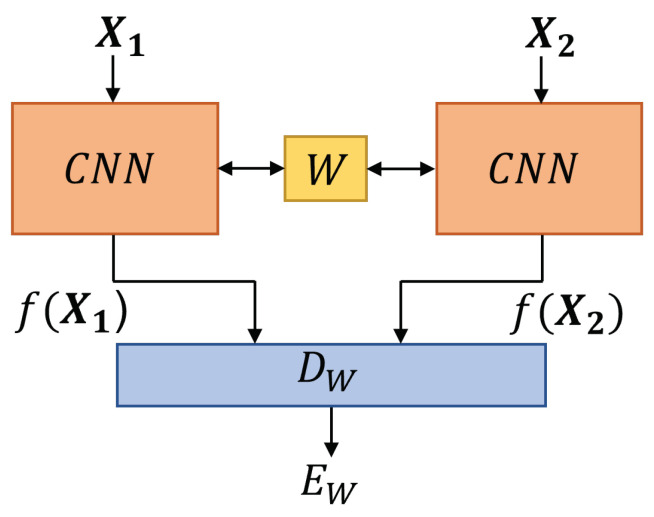
Schematic architecture of a Siamese network, adapted from [22]. Where $X_{1}$ and $X_{2}$ are the inputs for identical CNNs with a shared weight *W* optimized to minimize the loss function (see Equation (3)). The distance function $D_{W}$ is calculated as Euclidean distance between *f*(X${}_{1})$ and *f*(X${}_{2})$. Thus $E_{W}$ can measure the similarity between $X_{1}$ and $X_{2}$.

$X_{1},X_{2}\in R^{40\times 40\times 3}$ are multidimensional inputs of identical network structure (see Figure 4), which are analyzed with $W\in R^{m\times n}$ and represents a shared weight. The inputs include three channels (major strain, minor strain and thickness) with the height (rows) and width (columns) of 40. The network’s outputs, $f(X_{1})$ and $f(X_{2})$ are normalized with the L2 norm. In the following step, both outputs are pair-wise evaluated with a distance function ($D_{W}$), based on Euclidean distance between both outputs ($f\left(X_{1}\right),f\left(X_{2}\right)$). The distance function $D_{W}$ is defined as follows:

(1) $$D_{W}\left(X_{1},X_{2}\right)=||f\left(X_{1}\right)-f\left(X_{2}\right)|{|}_{2}$$

The training aims to minimize an objective function L known as a loss function (error function) by finding the optimal W [23]. *Y* is a binary label mapped to the pair of inputs. When both inputs are similar, the label is Y=0, in case $X_{1}$ and $X_{2}$ are deemed dissimilar, then label is defined as Y=1. In general ${\left(Y,X_{1},X_{2}\right)}^{i}$ is defended as the *i*-th labeled sample pair [24].

(2) $$L\left(W\right)=\sum_{i=1}^{P}\;L\left(W,{\left(Y,X_{1},X_{2}\right)}^{i}\right)$$

$L_{S}$ and $L_{D}$ are partial loss functions, the first referring to similar pairs and the second to those described as dissimilar. The number of pairs that are trained is defined as *n*. As mentioned, the goal is to minimize the loss function. Thus, the function $D_{W}$ must assume small values for similar pairs and high values for dissimilar pairs [24]. $E_{W}$ can be defined as a function (see Equation (5)) that measures the compatibility or similarity between the inputs $X_{1}$ and $X_{2}$ [22]. The resulting function is described as in Equation (3).

(3) $$L\left(W,{\left(Y,X_{1},X_{2}\right)}^{i}\right)=\left(1-Y\right)L_{S}\left(D_{W}^{i}\left(X_{1},X_{2}\right)\right)+YL_{D}\left(D_{W}^{i}\left(X_{1},X_{2}\right)\right)$$

(4) $$L_{S}\left(W,X_{1},X_{2}\right)=\frac{1}{2}{\left(D_{W}\right)}^{2}$$

(5) $$L_{D}\left(W,X_{1},X_{2}\right)=\frac{1}{2}max{\left\{\left(0,m-D_{W}\right)\right\}}^{2}$$

The dissimilar pairs are calculated into the loss function if those are located inside the radius *m* around the function f(X) [24].

#### 3.1.3. Clustering and Postprocessing

After the training phase, the network learns to separate two extreme states, the first one, the homogeneous, without necking sequence, and the second one, a sequence with a fracture. The whole forming process and the sequences between the two states (homogeneous and near of fracture) are unknown, but the model is able to extract discriminative features from the complete sequences. The principal component analysis was used to find the onset of necking and cluster the data into homogeneous and necked areas. Principal component analysis (PCA) is a technique to analyze data with a high number of features (dimensions) with the aim to reduce the features and extract the important information [25]. The Student’s t Mixture Models [26] was used to cluster the features into homogeneous, transition and inhomogeneous classes in order to detect the onset of the necking.

#### 3.1.4. Evaluation

To evaluate the results, a leave-one-material-out (LOMO) cross-validation (CV) has been applied. The NN trained on three materials, and for validating the fourth one was used in order to examine how well the model can predict the onset of necking on an unknown (that the network has never seen before) material. This procedure was conducted in the inference four times, where the validation was carried out by each material.

**Figure 5:**
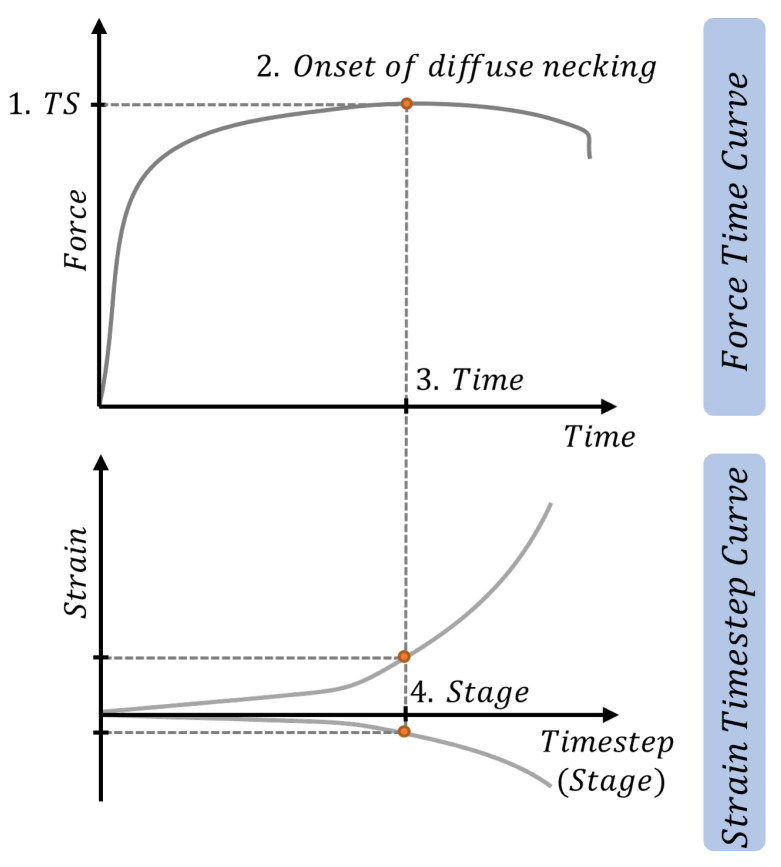
Ground truths investigation procedure based on force-time curves and strain-timestep curves.

The following procedure, shown in Figure 5, was applied to evaluate the results. During tensile tests, the force and time data was collected. On the point of maximal stress (1), the onset of diffuse necking can be detected (2). Step (3) can be used to determine stage (4) in Strain-Time step data so the values (major strain and minor strain) can be noted.

The error between the label (strain from strain-timestep curve) and prediction (output from the network) is calculated as follows:

The error between the label (strain from the strain-timestep curve) and prediction (output from a network) is conducted in accordance with Equation (6). This equation has been defined for this work to describes the difference between the two values.

(6) $$\begin{array}{c} \Delta \epsilon =|\epsilon_{\;label}-\epsilon_{\;prediction}| \end{array}$$

### 3.2. Image-Based Approach

As input, the image-based approach uses the images of the video sequence recorded by the stereo camera system during the forming process. Therefore, an evaluation for the input data set by DIC is not necessary. The classification output of the method refers to the time point at which the local necking was detected. The data processing of the image-based approach provides a modified procedure for preprocessing and classification compared to the method developed by Jaremenko et al. [16]. Validation of the method is performed for comparability using the previously proposed LOMO-CV approach. The evaluation is carried out using the probabilistic FLC explained in previous work [17], as well as on an evaluation approach adapted for the method.

#### 3.2.1. Preprocessing

**Figure 6:**
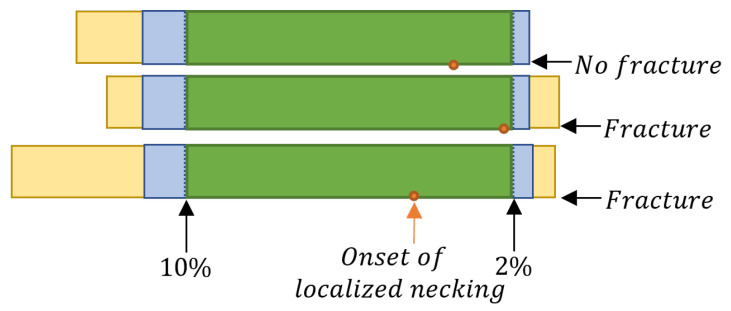
The blue section of the data (10% of the beginning and 2% of the end) was used in the image-based approach for the training. In the inference the whole, unknown data sets (yellow, blue and green) were applied. The point of the onset of localized necking was determined with the cross-section method.

Twenty-four specimens were elongated to the fracture, and additionally, five records of the tensile tests ended shortly after the onset of localized necking. In the first step, the data sets were cropped at the last stage before fracture to increase the amount of the data (29 specimens). Next, the data length was normalized to the the shortest data set and cropped to the stages at the beginning of the recording. The images have been channel-wise normalized with their mean and standard deviation. For the training the size of the sections, homogeneous and inhomogeneous, are specified. In the beginning, 10% of the data was assigned to the homogeneous class, and the data with a minimum margin of 2% from the end of the (blue in Figure 6) sequence was considered as inhomogeneous class. The preprocessing method for three exemplary specimens is shown in Figure 6.

#### 3.2.2. Training

**Figure 7:**
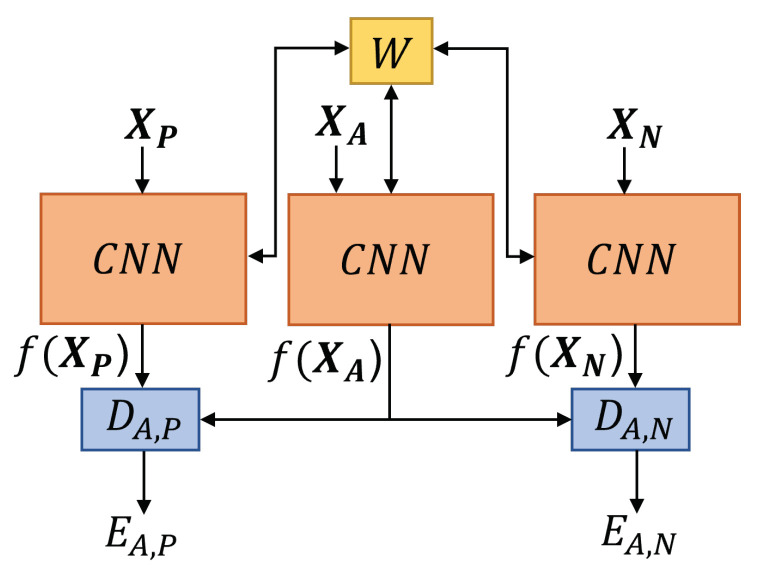
Schematic architecture of the network, where $X_{A},X_{P},X_{N}$ are the inputs (anchor, positive and negative) of the network, which trains with a shared weight. This weight is used by the CNN for the optimization by minimization of the loss function. The distance functions are calculated as Euclidean distance between $f\left(X_{A}\right)-f\left(X_{P}\right)$ and $f\left(X_{A}\right)-f\left(X_{N}\right)$.

$X_{A},X_{P},X_{N}\in R^{400\times 400\times 2}$ are multidimensional inputs of the network structure (see Figure 7), where $X_{A}$ represents the anchor input, $X_{P}$ a positive input of the same class as $X_{A}$ and $X_{N}$ is the negative input different to the class $X_{A}$. Each input consists of two channels (images of two different cameras) and each image has the height and width of 400 px. The inputs are analyzed through the CNN (DenseNet) [27]) with a shared weight W. The outputs of the network $f(X_{A})$, $f(X_{P})$ and $f(X_{N})$ are normalized by the L2 norm. $D_{A,P}$ and $D_{A,N}$ are distance functions (see Equations (7) and (8)), where the first one describes the Euclidean distance between normalized $f(X_{A})$ and $f(X_{P})$ and the second one the Euclidean distance from the normalized $f(X_{A})$ to $f(X_{P})$.

(7) $$D\left(X_{A},X_{P}\right)=||f\left(X_{A}\right)-f\left(X_{P}\right)|{|}_{2}$$

(8) $$D\left(X_{A},X_{N}\right)=||f\left(X_{A}\right)-f\left(X_{N}\right)|{|}_{2}$$

As well as in the numerical approach the goal of training is to minimize an objective function. In this architecture a triplet loss [28] is used, see Equation (10). α describes the margin (in this work 1) between the anchor and positive input as well as anchor and negative input. The maximum of the subtraction of the distance functions plus margin and 0 is used for optimization. If the difference between both distances is smaller than the negative margin, the optimization is not taking place for the particular sample.

(9) $$L\left(W\right)=\sum_{i=1}^{n}\;L\left(W,\left(\alpha ,X_{A},X_{P},X_{N}\right)\right)$$

(10) $$L\left(W,\alpha ,X_{A},X_{P},X_{N}\right)=\max\left(D\left(X_{A},X_{P}\right)-D\left(X_{A},X_{N}\right)+\alpha ,0\right)$$

#### 3.2.3. Postprocessing and Evaluation

**Figure 8:**
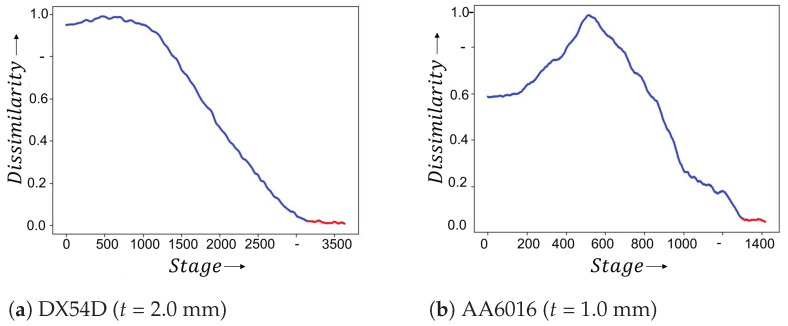
After the training the time point of onset of the localized necking (red) is detected when the prediction curve converges. The model compares all stages (sequences) with the last one and predicts the normalized dissimilarity (Euclidean distance in the embedding). The point of convergence is located, where the value does not deviate more than 5% compared to the average of the last 5% of the stages.

After the training phase, the network learns to separate the two extreme states, the first one, the homogeneous, without localized necking sequence, and the second one, a sequence near of the fracture. The whole forming process and the sequences between the two states (homogeneous and near of fracture) are unknown, but the model is able to extract discriminative features from the entire sequences. In this work for the image-based approach two evaluation methods are proposed. In the first method after the inference, the sequence, where the predicted curve converges, has to be detected. Figure 8a,b show two examples (for DX54D, *t* = 2.0 mm and AA6016), where the convergence (onset of localized necking) is determined. The second evaluation technique contains a probabilistic analysis. It is investigated with which probability an image belongs to the necking class.

To evaluate the results of these two methods the same technique (LOMO-CV) as in the numerical approach was applied. The LOMO-CV carried out on three materials. The fourth one was used for validation of the accuracy of the model in order to investigate if it can predict the onset of necking on an unknown material. This procedure was conducted in the inference four times, where the validation was carried out with each material.

## 4. Results

### 4.1. Numerical Approach

**Figure 9:**
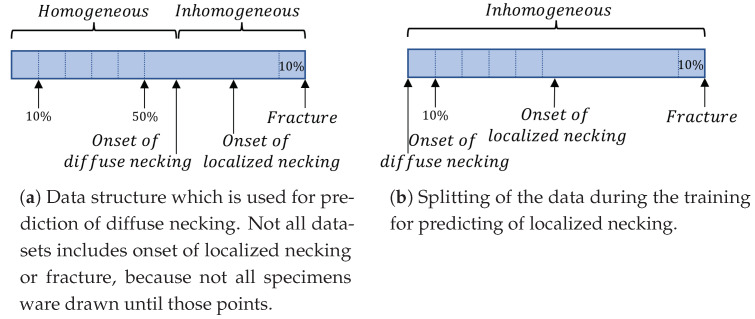
For the experiments different data-splitting setups ware used. For predicting of the diffuse necking the whole data.

As described in Section 3 during the training phase the network receives a subsequence containing the homogeneous forming phase and a subsequence where damage to the specimen is already observed. During training, Jaremenko et al. [16] calculated the difference in strain data and determined the optimal network on this basis. For tensile tests, no satisfying result can be obtained with the initial partitioning of the data, accordingly different combinations of the data splitting (Figure 9a,b) were tested. A dependence of this partitioning against the detected time points representing the transition between the forming phases was found.

#### 4.1.1. Prediction of Diffuse Necking

**Figure 10:**
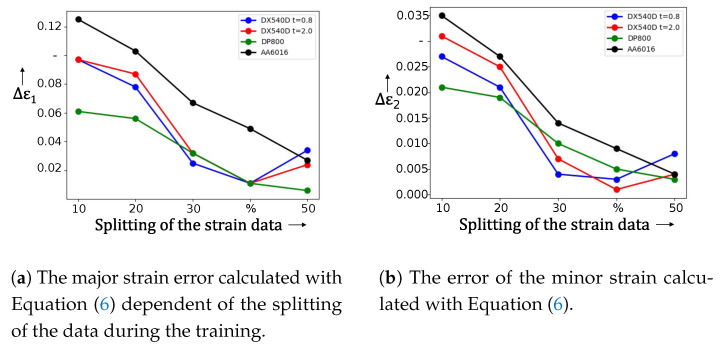
Detection results of the diffuse necking onset where the error of $\epsilon_{1}$ and $\epsilon_{2}$ are shown. The best constellation was achieved with 40% of the homogeneous data.

The more the network trained from the homogeneous phase, the larger the relative error was. For this reason, the 10% of fracture data was fixed and tested further with different splitting combinations of the homogeneous part from 10% to 50% (more than 50% would cover the diffuse necking time point). The results are shown in Figure 10a for major strain and in Figure 10b for minor strain. For DX54D, the best combination was achieved with a distribution of homogeneous data of 40%. For major strain for DX54D an error of $\Delta \epsilon_{1}=0.011$ was reached, for minor strain a mean $\Delta \epsilon_{1}=0.002$ was achieved. For steel DP800 we achieved the best results ($\Delta \epsilon_{1}=0.011$ and $\Delta \epsilon_{2}=0.005$) with the split size of 50%. The aluminium alloy also performed best with the distribution of 50% ($\Delta \epsilon_{1}=0.027$ and $\Delta \epsilon_{2}=0.004$). In the second experiment, only the data, which includes tests drawn until fracture, was used. Again the different splitting combinations were investigated. DX54D *t* = 0.8 mm and DP800 remained relatively stable for all combinations. AA6016 has shown a dependency for 10% and 20%, and from 30%, the behavior can be described as robust against changes in the distribution of the data. Validation of DX54D *t* = 2.0 mm resulted in significant deviations regarding strain values on the point of tensile strength.

**Figure 11:**
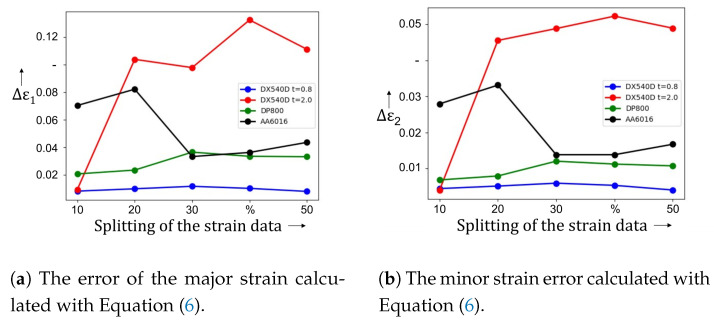
Detection results by using only the data, including tension rods which were elongated to the fracture. The DX54D *t* = 0.8 mm and DP800 show robustness against changes in the splitting of the training data. AA6016 is also stable above 20% of the homogeneous data. DX54D *t* = 2.0 mm resulted with large deviations above 10%.

In the next experiment only specimens whose elongation extended to fracture were used. Figure 11a for major strain and in Figure 11b for minor strain show the results achieved with the unsupervised classification method. DX54D *t* = 0.8 mm for $\Delta \epsilon_{1}$ ranges between 0.008 and 0.018 and for $\Delta \epsilon_{2}$ between 0.004 and 0.008. DP800 resulted with values within 0.021 and 0.034 for $\Delta \epsilon_{1}$ and 0.007 to 0.012 for $\Delta \epsilon_{2}$. The range of AA6016 show following values: 0.033 and 0.082 for $\Delta \epsilon_{1}$ as well as 0.013 and 0.033 for $\Delta \epsilon_{2}$. DX54D *t* = 2.0 mm ranges between 0.009 and 0.133 for $\Delta \epsilon_{1}$ and between 0.004 and 0.005 for $\Delta \epsilon_{2}$.

Table 4 included quantitative results, the predicted strain values. An investigation of the influence of the number of data during the training was conducted. The best combination, in terms of the lowest error, regarding all examined materials is the 30–10% split. However, DX54D (*t* = 2.0 mm) shows a large deviation for this setup including all other distributions above 20%.

#### 4.1.2. Prediction of Localized Necking

As mentioned in Section 1, during the forming process different unstable phases can be observed: diffuse and localized necking, as well as fracture. In an additional experiment the local necking has been predicted. To detect the time point of the local necking, the data before diffuse necking has been removed. The same architecture has been used and as input served the data between the diffuse necking and fracture, see Figure 9a. This experiment resulted with large deviation of the strain compared to [7], see Table 5. The FLC point for DX54D *t* = 0.8 mm by $\epsilon_{1}=0.664$, $\epsilon_{2}=-0.353$ provided by Kohl el al. [7] and the predicted point by $\epsilon_{1}=0.479$, $\epsilon_{2}=-0.206$ shown no alignment (for all materials and all splitting combinations). For DX54D *t* = 2.0 mm the CNN achieved an error of $\epsilon_{1}=0.509$, $\epsilon_{2}=-0.205$, for DP800 *t* = 1.0 mm $\epsilon_{1}=0.129$, $\epsilon_{2}=-0.046$ and for AA6016 *t* = 1.0 mm $\epsilon_{1}=0.191$, $\epsilon_{2}=-0.076$. Also in comparison to the values produced by Jaremenko et al. [16] based on Nakajima data, the results in this experiment show significant deviations.

### 4.2. Image-Based Approach

As mentioned in the previous Section 3.2, the CNN simultaneously obtains two images from the video sequence captured by the stereo camera system. The output of the network shows the membership probability and the similarity of a time step respectively towards the local necking phase. The obtained probabilities are discussed using the probalistic FLC approach, as well as the convergence criterion. To check the validity of the network, the concept of cross-validation is applied the same way as for the numerical approach.

**Figure 12:**
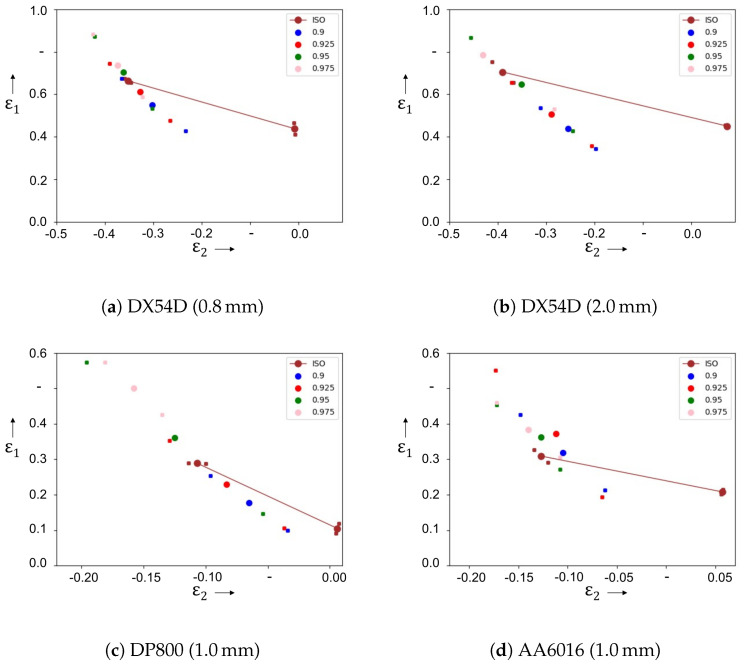
Probabilistic determination of onset of localized necking and the referencepoint calculated by the cross-section method (ISO 12004). The mean values are represent as dots and each dot refers to a threshold level.

The FLC support points were determined using different probability thresholds and compared with the support point defined using the cross section method, which is used as a reference (Figure 12).

The predicted support points of the material DX54D as well as DP800 are located on the same strain path as the reference support point. For the three materials, the smallest deviation from the forming limit determined according to the standard occurs at a probability threshold value of 0.95. Thereby, the error for the DX54D increases with increasing material thickness. Whereas a material thickness of 0.8 mm results in a slight overestimation of the forming limit compared to the reference, it is underestimated for the thicker material. For the dual-phase steel, higher degrees of deformation as well as a high deviation from the reference support point can be observed for the probability threshold value of 0.95. For the predicted forming limit under uniaxial strain of the aluminum alloy, deviations from the ideal uniaxial strain path occur depending on the probability threshold value. As for the DX54D and the DP800, the support point determined according to the probability threshold of 0.95 shows the smallest difference to the referencepoint. Overall, the results demonstrate a less accurate prediction ability than the currently established evaluation method.

For the similarity curve evaluated according to the convergence criterion, the support points of the forming limit curve are shown in Table 5. A good agreement with the respective reference support points can be demonstrated for the deep-drawing steel DX54D and the aluminum alloy AA6016. The forming limit of the dual-phase steel DP800 is predicted to be higher using the neural network than using the cross section method. The standard deviations for the predictions according to the machine learning method are higher than those using the standardized method.

## 5. Discussion

**Figure 13:**
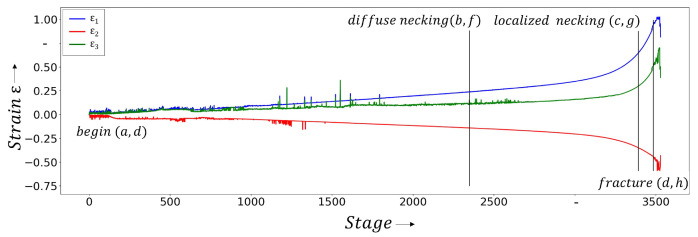
Strain values (major strain $\epsilon_{1}$, minor strain $\epsilon_{2}$ and thickness reduction $\epsilon_{3}$) from a ductile material (DX54D (*t* = 0.8 mm)) recorded during a tensile test. The images corresponding to the different states (begin, onset of diffuse necking, onset of localized necking and fracture) are shown in the figure below.

**Figure 14:**
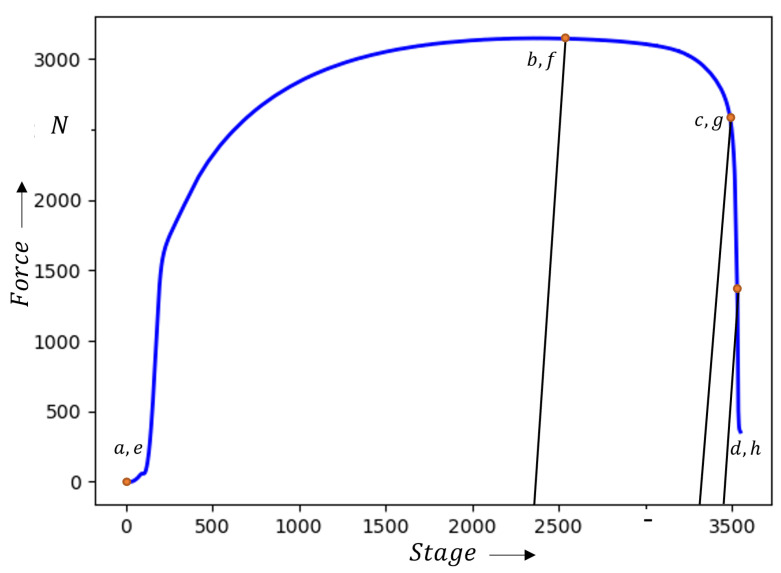
Force values (DX54D (*t* = 0.8 mm)) collected during a tensile test. The corresponding strain values are shown in the Figure 13 as well as the images in the Figure 15.

The results demonstrate that the detection of the local necking using the numerical approach based on data composed of the major-, minor strain-, and thickness-channels do not lead to any accordance with the reference support point. Instead, the approach is only able to reliably detect the onset of diffuse necking. This is due to the temporal development of major,-minor strain and thickness. Up to the onset of diffuse necking, the function of the three channels exhibits a proportional behavior over time (Figure 13) and the force values shown in Figure 14. After this point, the relationship between the respective attribute and time can be described approximately by an exponential function. The chosen method recognizes the change in behavior as a separating feature between the two classes of homogeneous forming and diffuse necking. Another feature to separate the diffuse and local necking was not found.

**Figure 15:**
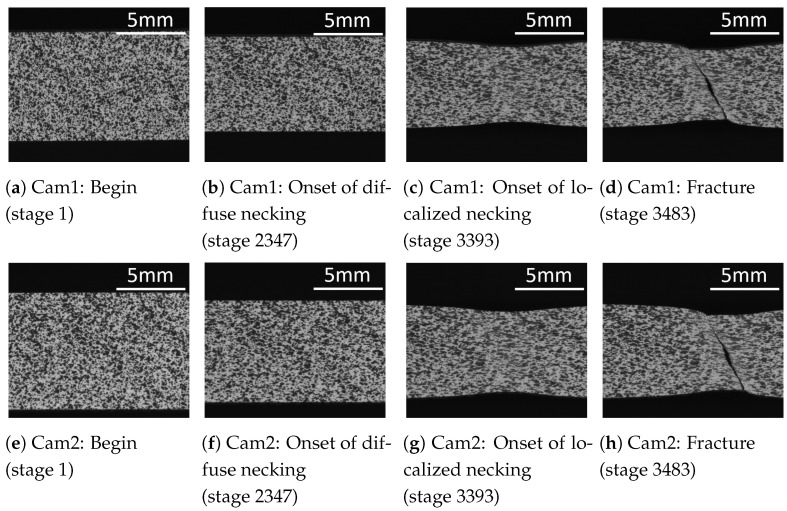
Different stages from two cameras (cams) show the grid pattern of the surface of a specimen (DX54D *t* = 0.8 mm).

The second approach uses the images from the stereo camera system as input data. These show a significant change in sample geometry and stochastic pattern at the onset of local necking (Figure 15). From the results, it can be inferred that these features are valid for the detection of the local necking. From the divergent results of the two approaches, it can be concluded that the representation of the data has a high relevance in determining the local necking using machine learning approaches.

To examine the generality of the features in combination with the evaluation methods, the LOMO-CV approach is applied and the transferability to unknown materials and material thicknesses is investigated. Since no results for the classification of the local necking can be obtained from the numerical approach, only the image-based approach is considered. For the probalistic based method, high deviations compared to the reference support point determined by the cross-section method are obtained. Especially for the dual-phase steel and aluminum alloy, a large error is observed. This may be due to the forming behavior of the materials, which has a much less distinct necking phase, like the deep drawing steel. Since no coverage of the forming limit predicted by the DL approach and the reference point can be observed, this approach does not show sufficient transferability to unknown data sets. If the convergence criterion, which is based on the similarity function derived from the network output, is used, the prediction of the support point of the forming limit curve according to the DL approach results in a good agreement with the reference support point for all materials. Thus, a robust and generalistic approach can be concluded. Nevertheless, the accuracy of the method is limited due to its high variance compared to the cross section method. The reason can be found in the low variability of the data set. It consists of only three materials and three different sheet thicknesses, which were formed under constant test conditions and geometries. The robustness of the prediction of the forming limit can be probably improved by increasing the range of variation of the data set by using a broader selection of materials, adding further sheet thicknesses or integrating tests with different combinations of test parameters.

Despite the lower accuracy, the approach has advantages compared to the cross-section method. Since the approach is based on the images of the stereo camera system, there is no need to calculate the strains of the complete video sequence using the DIC; instead, the approach refers to a single image of the sequence, which is analyzed for the corresponding strains in the following step. Due to the high computational efficiency of the neural network and the reduced effort of the processing steps to be performed in the Aramis software to determine the forming limit, there is a time saving, especially when high recording frequencies are applied. Furthermore, in contrast to the cross section method, the approach is independent of the location where the local necking occurs. Also, the occurrence of several strain maxima, as in the case of the aluminum alloy, does not influence the results.

## 6. Summary

In this study, we aimed to present an alternative, cost-efficient, and robust approach for determining the forming limit curve, with a specific focus on the support point under uniaxial tension. We developed two distinct approaches, one numerical and the other image-based:

- Numerical approach was adapted from Jaremeenko, utilizing a Siamese network based on VGG16 architecture. This network was trained using a contrastive loss function to predict the time point of instability based on three channels: major strain, minor strain and material thickness, primarily for detecting diffuse necking.
  – Furthermore, the method’s functionality remains unaffected by the specific location of diffuse necking.
  – However, it’s important to note that this approach showed dependencies related to the division of the training data.
- Image-based approach, an innovative image-based approach for detecting localized necking was introduced. The architecture includes a Siamese network with DenseNet as a backbone trained through triplet loss. The network used as an input the images captured by a stereo camera system.
  – This approach replaces the need for labor-intensive strain calculations using Digital Image Correlation (DIC) with input from a stereo camera system. This shift greatly streamlines computational efficiency and reduces the effort needed to establish the support point on the forming limit curve.
  – The method demonstrates insensitivity to the formation of multiple strain maxima during the forming process. While the repeatability of this method is slightly lower than the standard method, it can be improved in future studies by diversifying the dataset.
  – In addition, the functionality of the method is independent of the location where the localized necking occurs.
  – For materials such as DX54D and AA6016, our method successfully predicted major strains in close agreement with ISO standards. However, it did not yield satisfactory results for DP800.
  – The repeatability of the method is less than the standard method, but can be improved for future studies by increasing the variance in the data set.

## Figures and Tables

**Table 1:** Properties of DX54D, DP800 and AA6016 with thickness ($t_{0}$), yield strength (YS), tensile strength (TS) and uniform elongation (UE).

Material	$t_{0}$ in mm	YS (MPa)	TS (MPa)	UE (%)
DX54D	0.8	173.8	316.6	22
DX54D	2.0	170.1	303.1	22
DP800	1.0	580.2	872.4	11
AA6016	1.0	154.4	261.1	17

**Table 2:** Chemical composition of DX54D, DP800 and AA6016 (% max. or range).

Material	C	Si	Mn	P	S	Ti	Al	Cr + Mo	Mg	Ni	Zn	Cu
DX54D	0.12	0.5	0.6	0.1	0.045	0.3	-	-	-	-	-	-
DP800	0.15	0.42	2.06	0.008	0.002	-	0.57	0.408	-	-	-	-
AA6016	-	0.5	0.25–0.6	-	-	-	-	-	0.1	0	0.15	0.2

**Table 3:** Materials and the number of all tested specimens *n*, $n_{L}$ number of specimens drawn until localized necking and $n_{R}$ number of the specimens elongated to fracture.

Material	*n*	$n_{L}$	$n_{R}$
DX54D (0.8 mm)	36	7	4
DX54D (2.0 mm)	39	7	7
DP800	38	8	6
AA6016	39	7	7

**Table 4:** Results showing a comparison between measured and predicted TS (tensile strength), UE (uniform elongation) and the strain values at the onset of diffuse necking. TS has been measured with the Z100 universal testing machine. The Aramis system provides strain values. Predicted results refer to training with data (splitting 20%/10%), including the specimens elongated to fracture.

Material and $t_{0}$	Measured				Predicted			
	** TS **	** UE **	** $\epsilon_{1}$ **	** $\epsilon_{2}$ **	** TS **	** UE **	** $\epsilon_{1}$ **	** $\epsilon_{2}$ **
DX54D (0.8 mm)	315	21	0.234	−0.137	315	21	0.222	−0.131
DX54D (2.0 mm)	299	22	0.237	−0.139	296	28	0.336	−0.183
DP800 (1.0 mm)	887	11	0.116	−0.039	884	12	0.149	−0.051
AA6016 (1.0 mm)	256	17	0.192	−0.077	255	12	0.158	−0.064

**Table 5:** Quantitative results of the detection of the onset of localized necking with the convergence criteria. This approach was trained, validated and tested on data (cross-validation), where 29 the specimens (see Table 1) ware elongated to the onset of localized necking.

Material and $t_{0}$	Prediction		ISO 12004	
	** $\epsilon_{1}$ **	** $\epsilon_{2}$ **	** $\epsilon_{1}$ **	** $\epsilon_{2}$ **
DX54D (0.8 mm)	0.659±0.143	−0.349±0.059	0.664±0.010	−0.353±0.007
DX54D (2.0 mm)	0.780±0.222	−0.412±0.094	0.705±0.049	−0.390±0.022
DP800 (1.0 mm)	0.478±0.139	−0.155±0.037	0.289±0.0014	−0.107±0.007
AA6016 (1.0 mm)	0.314±0.101	−0.117±0.041	0.309±0.018	−0.127±0.007

## Data Availability

Data is available upon request.
